# LncRNA MEG8 promotes deep vein thrombosis by sponging miR-296-5p to regulate human umbilical vein endothelial cells

**DOI:** 10.1186/s41065-025-00599-x

**Published:** 2025-11-11

**Authors:** Jiaqi Zhang, Menglan Li, Xingbang Na

**Affiliations:** 1 Nursing School, Zhejiang Chinese Medical University, Hangzhou, 310052 China; 2 Department of Hematology, Taikang Tongji (Wuhan) Hospital, Wuhan, 430050 China; 3 Department of Vascular Surgery, Ningbo Yinzhou No.2 Hospital, No.998 Qianhebei Road, Yinzhou District, Ningbo, 315192 China

**Keywords:** LncRNA MEG8, Deep vein thrombosis, MiR-296-5p, Human umbilical vein endothelial cells

## Abstract

**Aim:**

This study explored the diagnostic value and molecular mechanism of lncRNA MEG8 in deep vein thrombosis (DVT).

**Methods:**

This study included 120 patients with DVT and 100 healthy individuals as research subjects. Expression of lncRNA MEG8 and miR-296-5p in subjects’ serum were detected by RT-qPCR. Diagnostic ability of MEG8 for DVT occurrence analyzed by ROC curve. Logistic analysis was used to identify risk factors for DVT. Associations between MEG8 and other parameters were explored by Pearson correlation analysis. Migration, viability and apoptosis of transfected HUVECs were detected by Transwell method, CCK-8 assay and flow cytometry, respectively. In addition, inflammatory cytokines were detected using ELISA kits. The luciferase reporter assay established the interaction between MEG8 and miR-296-5p.

**Results:**

In patients with DVT, lncRNA MEG8 levels were significantly upregulated, and ROC curves showed high diagnostic ability. In addition, MEG8 was positively associated with TAT and D-dimer. In vitro experiments showed that overexpression of MEG8 inhibited HUVECs migration and viability, promoted apoptosis, and upregulated inflammatory factors such as IL-6, IL-1β, and TNF-α, while silencing of MEG8 showed the opposite effect. In addition, MEG8 regulated miR-296-5p expression by sponging it, and the dual luciferase reporter assay verified a direct interaction between them. Clinical samples revealed that serum miR-296-5p levels were diminished in DVT patients as well as negatively correlated with MEG8. Furthermore, miR-296-5p inhibitor reversed the role of MEG8 silencing on regulation of HUVECs migration, viability and inflammatory cytokines.

**Conclusion:**

This study revealed that MEG8 acts critically in DVT development through sponging miR-296-5p for the first time, providing a new molecular target for early diagnosis and targeted therapy of DVT.

**Supplementary Information:**

The online version contains supplementary material available at 10.1186/s41065-025-00599-x.

## Introduction

Deep vein thrombosis (DVT) is a syndrome in which pathological thrombosis in the deep veins leads to partial or complete obstruction of the blood vessels, which in turn leads to impaired venous return [1, 2]. It occurs more frequently in lower extremities such as femoral vein and is slightly more prevalent in males than females, as well as its prevalence increases substantially with age [3, 4]. After the first occurrence of DVT, some patients will develop post-thrombotic syndrome [5]. In severe cases, thrombus dislodgement leads to the occurrence of life-threatening pulmonary embolism [6]. All along, how to effectively prevent and treat DVT has been a widely concerned and urgent problem to be solved. Therefore, exploration of the molecular mechanism of DVT and search for biomarkers for early diagnosis and effective therapeutic targets have important clinical value.

Recent research has suggested that endothelial cell dysfunction acts as a key player in DVT formation [7]. As functional RNA molecules over 200 nt in length, long non-coding RNA (lncRNAs) have been confirmed as important regulatory actors in vascular biological processes. For example, lncRNA NORAD is highly expressed in DVT and is engaged in the progression of DVT by regulating proliferation and migration of human umbilical vein endothelial cells (HUVECs) [8]. LncRNA ANRIL affects angiogenesis and thrombosis by regulating miR-99a and miR-449a [9]. Previous reports demonstrated that lncRNA MEG8 acts as a regulator in several diseases and takes part in the regulation of vascular function [10]. Zhang et al. identified an increase of MEG8 in gestational diabetes and exhibits the ability to predict kidney injury [11]. Ma et al. observed that MEG8 inhibited hemangioma endothelial cell proliferation via modulating miR-497-5p/NOTCH2 axis [12]. In addition, MEG8 contributes to the alleviation of vascular injury by inhibiting hypoxia-induced vascular smooth muscle cell hyperproliferation, migration and inflammation via modulation of miR-195-5p/RECK axis [13]. More importantly, it was found that elevated MEG8 levels predict venous thromboembolism in ovarian cancer patients [14]. Nevertheless, the regulatory role of lncRNA MEG8 in HUVECs is unclear, and the molecular mechanisms especially in DVT remain to be explored.

Several studies have shown that lncRNAs regulate disease progression by sponging miRNAs. Guo et al. reported that lncRNA MEG8 promotes non-small cell lung cancer progression via targeting miR-15a-5p and miR-15b-5p [15]. MEG8 regulates angiogenesis and alleviates cerebral ischemia after ischemic stroke by targeting the miR-130a-5p/VEGFA pathway [16]. Existing research suggests that the proliferation, migration and angiogenesis of HUVECs may be associated with miR-296-5p differential expression [17]. Moreover, Pan et al. found that miR-296-5p was reduced in DVT patients and may be a potential diagnostic molecule [18]. Bioinformatics predictions suggested that MEG8 may contain complementary binding sites to miR-296-5p, implying that it may be involved in the DVT process through a competitive endogenous mechanism. However, up to now, there is no experimental evidence to support the role of MEG8 and miR-296-5p regulatory networks in modulating cellular function and inflammatory factor secretion in HUVECs, and their value in clinical DVT diagnosis has not been systematically assessed.

In our study, the molecular mechanism by which MEG8 regulates HUVECs by sponging miR-296-5p was verified through case-control analysis and in vitro experiments. This study offers novel molecular markers for early diagnosis of DVT and provides theoretical basis for targeted intervention of lncRNA-miRNA network in the prevention and treatment of thrombotic diseases.

## Materials and methods

### Collection of samples

Approval was granted by the Ethics Committee of Ningbo Yinzhou No.2 Hospital, and all subjects signed informed consent. This study enrolled 120 patients with DVT admitted to our hospital from 2021 to 2023, and 100 volunteers who had a physical examination around the period were selected as controls. Inclusion criteria for DVT patients were ultrasound doppler or venography-confirmed DVT of the lower extremities, a significantly elevated plasma D-dimer level, and no previous history of thrombotic disease. The inclusion criteria for controls were matching age and gender with DVT patients, no personal or family history of thrombosis, and normal coagulation function. Exclusion criteria for both groups were those suffering from malignant tumors, primary vascular diseases, circulatory diseases, and immunodeficiency diseases, as well as those using antiplatelet, anticoagulant, and thrombolytic drugs. The age, gender and body mass index (BMI) of study subjects were collected through case retrieval. Venous blood was drawn from patients in the fasting state in the early morning and sent for testing of low density lipoprotein cholesterol (LDL-C), high density lipoprotein cholesterol (HDL-C), white blood cell (WBC), thrombin -antithrombin (TAT), and D-dimer.

### Cell culture and transfection

Human umbilical vein endothelial cells (HUVECs) were purchased from ScienCell Research Laboratories in the United States. They were cultured in DMEM medium (Sigma-Aldrich, German) containing FBS, endothelial cell growth additives and dual antibodies at 37 °C in a 5% CO^2^ humidified incubator. To mediate the expression of lncRNA MEG8 and miR-296-5p, si-MEG8, oe-MEG8, miR-296-5p inhibitor, miR-296-5p mimic as well as their negative controls were constructed and synthesized by GenePharma in Shanghai, China. When HUVECs reached logarithmic growth phase, cells were transfected using Lipofectamine 2000 (Invitrogen, USA).

### RNA extraction and RT-qPCR

The following morning, 4 mL of fasting venous blood was taken and centrifuged at 1500 g for 10 min to separate the supernatant serum. The serum samples were stored at −80°C for subsequent RNA extraction. Total RNA was extracted from the above-mentioned serum or cells using Trizol reagent (Thermo Fisher, USA). First-strand cDNA synthesis was performed with the PrimeScript RT kit (TAKARA, Japan). RT-qPCR assays were conducted using SYBR Green quantitative PCR premix (Roche, Switzerland) on ABI 7500 Real-Time PCR System (Applied Biosystems, USA). The thermocycling conditions for detecting MEG8 were as follows: Initial denaturation at 95 °C for 15 min; followed by 40 cycles at 95 °C for 15 s, 60 °C for 15 s and 72 °C for 15 s. The thermocycling conditions for detecting miR-296-5p were as follows: Initial denaturation at 95 °C for 15 min; followed by 30 cycles at 95 °C for 15 s, 60 °C for 15 s and 72 °C for 15 s. The endogenous control for MEG8 was GAPDH, and for miR-296-5p was U6. Relative gene expressions were calculated by the 2^−ΔΔCt^ method. The specific primers were as follows: MEG8 forward, 5’- CCTCAGTATCCTGCGAGCTG-3’ and reverse, 5’-CTCCTGAGGCTTTCATGCCA-3’; miR-296-5p forward, 5’-AGGGCCCCCCCUC-3’ and reverse, 5’-GTGCAGGGTCCGAGGT-3’; GAPDH forward, 5’-CATCAACGGGAAGCCCATC-3’ and reverse, 5’-CTCGTGGTTCACACCCATC-3’; U6 forward, 5’-CTCGCTTCGGCAGCACA-3’ and reverse, 5’-AACGCTTCACGAATTTGCGT-3’.

### Cell migration assay

The cell suspension was added to the upper chamber of the Transwell (BD, USA) and the lower chamber was filled with medium containing FBS. It was incubated in an incubator at 37 °C, 5% CO^2^ for 24 h. After removing the cells from the upper chamber, they were fixed with paraformaldehyde solution for 15 min and stained with crystal violet for 30 min. The stained cells were observed by a microscope (Nikon, Japan).

### Cell viability assay

Cell viability was assayed using the CCK-8 kit (Dojindo, Japan). HUVECs were digested with trypsin and resuspended in complete medium. The cell concentration was adjusted to 4 × 10^4^ cells/mL, and the cells were seeded at 100 µL/well in 96-well plate. At 0, 24, 48, and 72 h of cell culture, 10 µL of CCK8 solution was added to the corresponding wells and the incubation was continued for 2 h. Absorbance at 450 nm was recorded with a microplate reader (Molecular Devices, USA).

### Apoptosis assay

Cell apoptosis was detected using the Annexin V-APC/7-AAD kit (Biolegend, USA). Transfected HUVECs were digested with trypsin and collected. The cell suspension was prepared using Annexin V Binding Buffer and transferred to a flow tube. Subsequently, APC Annexin and 7-AAD Viability Staining Solution were added, and the staining was protected from light for 15 min. A flow cytometer (BD, USA) was used for detection and data analysis.

### Inflammatory cytokines assay

Supernatants of transfected cells were collected and inflammatory cytokines IL-6, IL-1β and TNF-α levels were assayed using ELISA kits (Abcam, UK).

### Luciferase reporter assay

The 3’-UTR sequence or mutant sequence of MEG8 containing the miR-296-5p binding sites were cloned into the pmirGLO vector (Promega, USA). The luciferase reporter plasmids (MEG8-WT, MEG8-MUT) were synthesized by GenePharma (Shanghai, China). The luciferase reporter plasmids and miR-296-5p inhibitor, miR-296-5p mimic or their negative control were co-transfected into HUVECs using Lipofectamine 2000 (Invitrogen, USA). Cells were obtained after 48 h and analyzed for luciferase activity using luciferase reporter assay system (Promega, USA) with Renilla as an internal reference.

### Statistical analysis

Data statistics and analyses were presented using GraphPad 9.0 and SPSS 27.0 software. The data is expressed as mean ± SD. The t-test was used to compare the means between the two groups, and one-way ANOVA was applied to analyze the significance of the differences among three or more groups. The ROC curve was utilized to analyze the accuracy of MEG8 in diagnosing DVT. Risk factors associated with the occurrence of DVT were assessed by logistic regression analysis. Pearson correlation analysis was employed to evaluate association of MEG8 with clinically relevant indicators and miR-296-5p. *P* < 0.05 were considered statistically significant.

## Results

### Clinical characteristics of subjects

The basic clinical characteristics of the 120 patients with DVT and 100 healthy individuals in the study were summarized in Table 1. The results revealed that by comparing the basic information of the two groups, there was no significant difference between them in terms of age, gender, BMI, LDL-C and HDL-C, which proved the comparability of the two groups. However, WBC, TAT and D-Dimer were considerably higher in DVT group (*P* < 0.01).

**Table 1:** Clinical features of the subjects

	Healthy *n* = 100	DVT *n* = 120	*P* value
Age (years)	60.89 ± 10.83	62.35 ± 10.12	0.303
Gender (male/female)	56/44	72/48	0.551
BMI (kg/m^2^)	24.51 ± 3.02	24.16 ± 3.98	0.472
LDL-C (mmol/L)	3.22 ± 0.78	3.36 ± 0.67	0.142
HDL-C (mmol/L)	1.52 ± 0.23	1.48 ± 0.10	0.095
WBC (×10^9^/L)	5.09 ± 0.85	5.37 ± 0.66	0.005
TAT (µg/L)	2.32 ± 0.99	6.66 ± 1.81	< 0.001
D-Dimer (mg/L)	0.43 ± 0.05	4.55 ± 0.71	< 0.001

*DVT* Deep Vein Thrombosis, *BMI* Body Mass Index, *LDL-C* Low Density Lipoprotein Cholesterol, *HDL-C* High Density Lipoprotein Cholesterol, *WBC* White Blood Cell, *TAT* Thrombin-Antithrombin

### Ability of MEG8 to diagnose DVT

**Fig. 1:**
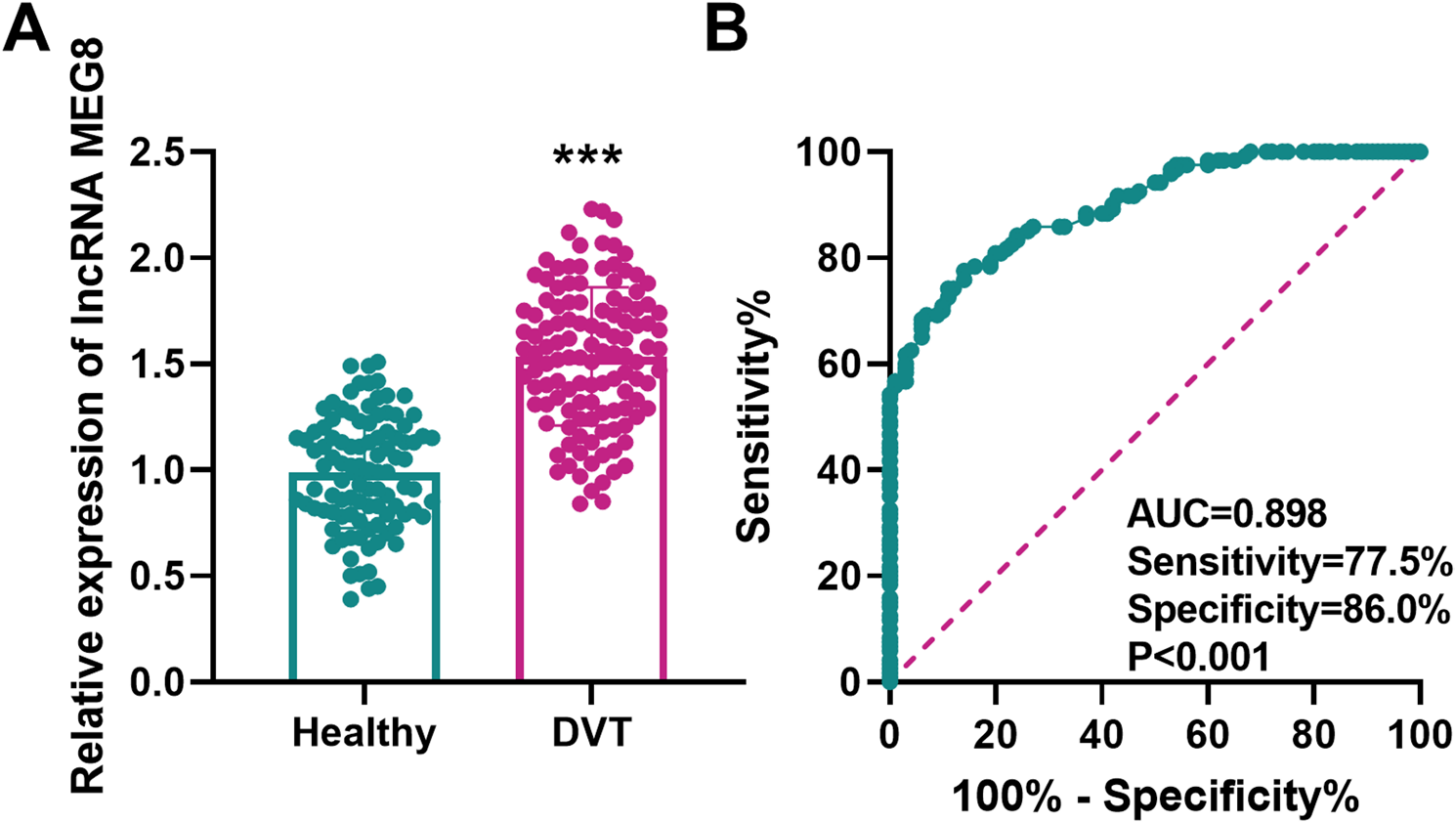
Ability of lncRNA MEG8 to diagnose DVT. **A** Expression of lncRNA MEG8 in the serum of DVT patients and healthy individuals. **B** ROC curve for the diagnosis of DVT occurrence by lncRNA MEG8. ***P < 0.001

To analyze the diagnostic function of MEG8 in DVT, we examined serum MEG8 levels in DVT patients and healthy individuals. RT-qPCR results suggested that MEG8 was significantly elevated in DVT patients (Fig. 1A). ROC curve analysis revealed a high diagnostic capacity of MEG8, with an area under the cure (AUC) of 0.898, a cut-off value of 1.28, a sensitivity of 77.5% and a specificity of 86.0% (*P* < 0.001, Fig. 1B). In addition, logistic regression analysis indicated that, apart from TAT (*P* = 0.003) and D-Dimer (*P* = 0.001), MEG8 (*P* < 0.001) was also an independent risk factor for DVT occurrence (Table 2).

**Table 2:** Logistic analysis of risk factors on DVT occurrence

	OR	95% CI	*P* value
LncRNA MEG8	12.350	5.901–25.847	< 0.001
Age (years)	1.907	0.939–3.872	0.074
Gender (male/female)	1.853	0.923–3.718	0.083
BMI (kg/m^2^)	0.633	0.318–1.260	0.193
LDL-C (mmol/L)	1.641	0.821–3.279	0.161
HDL-C (mmol/L)	0.536	0.269–1.065	0.075
WBC (×10^9^/L)	1.722	0.870–3.409	0.119
TAT (µg/L)	2.901	1.452–5.794	0.003
D-Dimer (mg/L)	3.371	1.617–7.027	0.001

*DVT* Deep Vein Thrombosis, *OR* Odds Ratio, *95% CI* 95% confidence interval, *BMI* Body Mass Index, *LDL-C* Low Density Lipoprotein Cholesterol, *HDL-C* High Density Lipoprotein Cholesterol, *WBC* White Blood Cell, *TAT* Thrombin-Antithrombin

### Correlation of MEG8 with clinical indicators of DVT

**Fig. 2:**
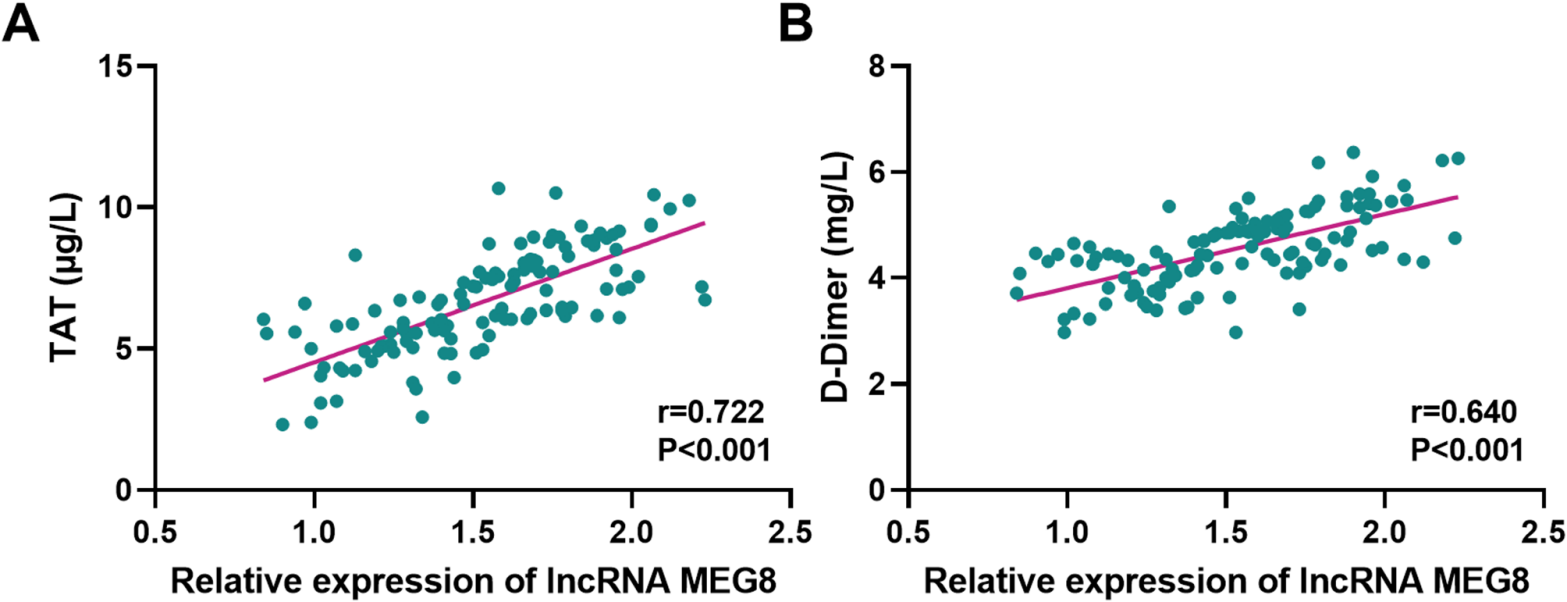
Correlation of lncRNA MEG8 with clinical indicators of DVT. LncRNA MEG8 levels in serum of DVT patients were positively correlated with (**A**) TAT and (**B**) D-Dimer

To assess the association between MEG8 levels and clinical indicators in DVT patients, we conducted the Pearson correlation analysis. The results as shown in Figs. 2A and B demonstrated a significant positive correlation of MEG8 with TAT (*r* = 0.722, *P* < 0.001) and D-Dimer (*r* = 0.640, *P* < 0.001) in DVT patients.

### Effect of MEG8 on migration, viability, apoptosis and inflammatory cytokines in HUVECs

**Fig. 3:**
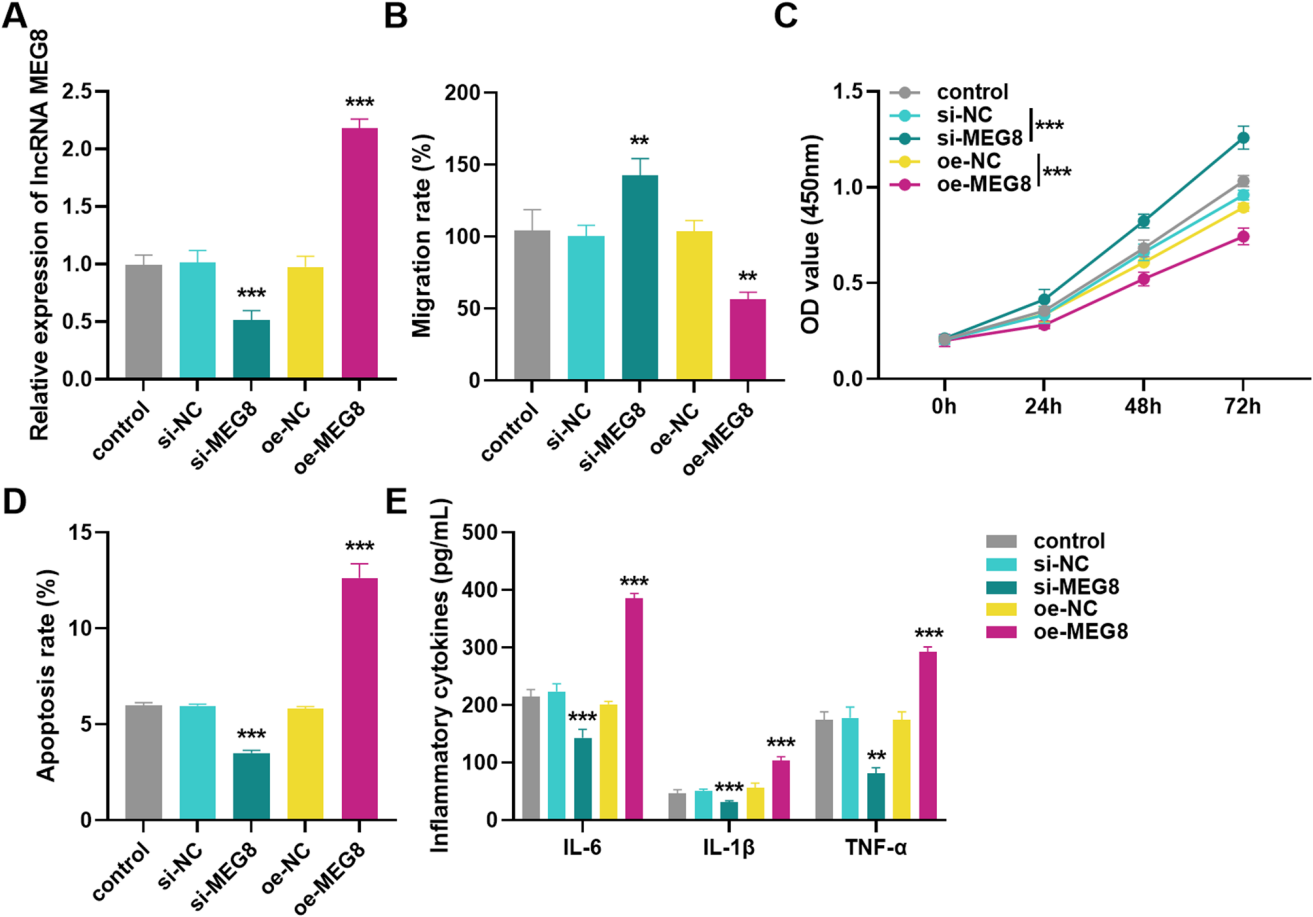
Effect of lncRNA MEG8 on HUVECs. Effects of silencing or overexpressing lncRNA MEG8 on (**A**) MEG8 levels, (**B**) migration, (**C**) cell viability, (**D**) apoptosis, (**E**) IL-6, IL-1β and TNF-α in HUVECs. **P < 0.01, ***P < 0.001

In the research of DVT, HUVECs are usually used as in vitro cell models. To analyze the effect of MEG8 on DVT development, we validated it by transfecting HUVECs. The expression outcome of silencing or overexpression of MEG8 was shown in Fig. 3A, which was dramatically reduced after silencing MEG8 and increased after overexpression of MEG8, proving that the successful transfection could be used for subsequent experiments. Cel l migration assays demonstrated that silencing MEG8 promoted HUVECs migration, whereas overexpression of MEG8 inhibited it (Fig. 3B). Cell viability assays revealed that silencing MEG8 markedly enhanced the viability of HUVECs, whereas overexpression of MEG8 reduced the viability (Fig. 3C). The apoptosis outcome showed an opposite trend, silencing MEG8 inhibited apoptosis, while overexpression of MEG8 promoted it (Fig. 3D). In addition, ELISA experiments were performed to detect inflammatory cytokines, indicating that down-regulation of MEG8 decreased IL-6, IL-1β, and TNF-α levels, whereas up-regulation of MEG8 elevated their levels (Fig. 3E).

### MEG8 sponges to miR-296-5p

**Fig. 4:**
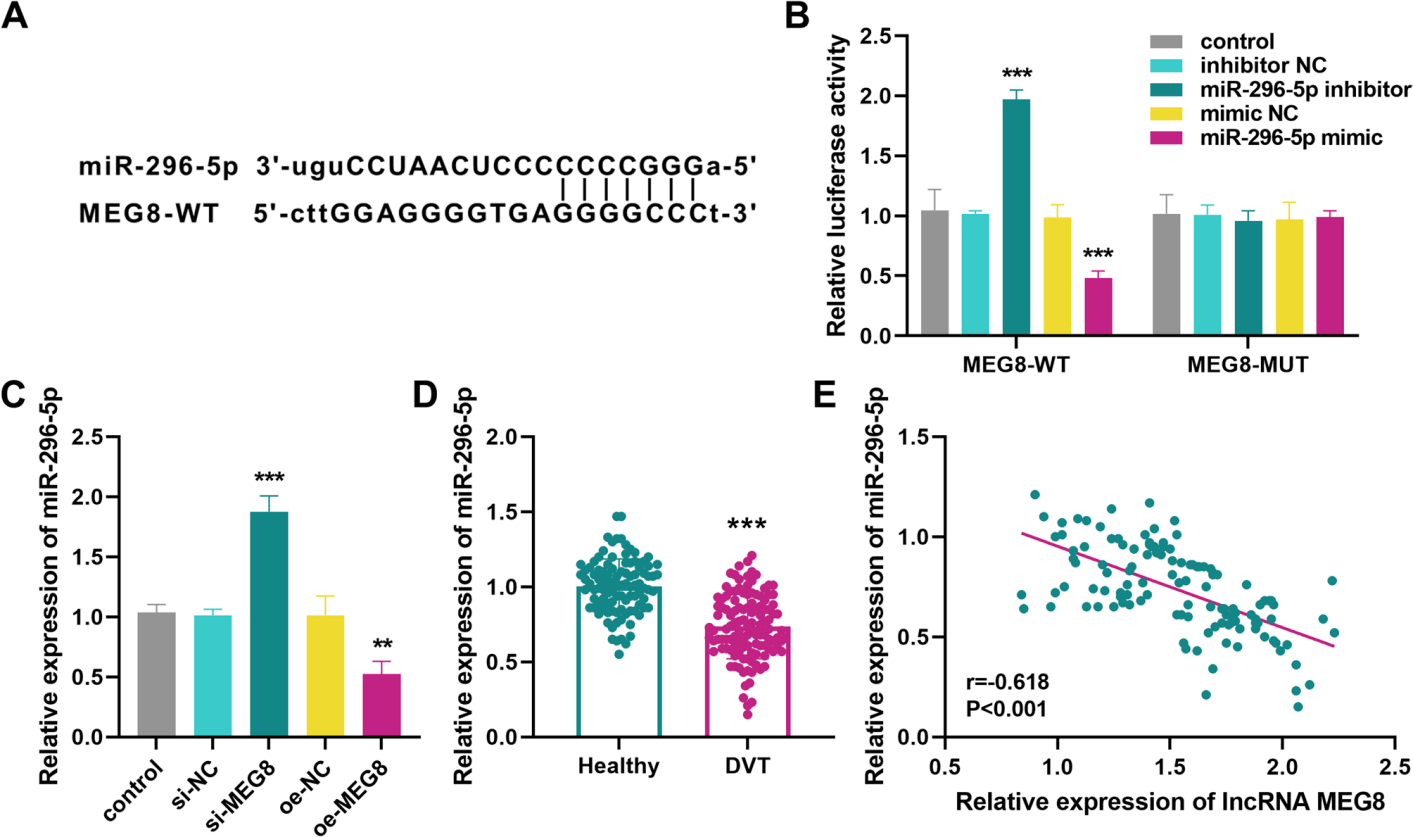
lncRNA MEG8 sponges miR-296-5p. **A** Binding sites of lncRNA MEG8 to miR-296-5p. **B **Relationship between lncRNA MEG8 and miR-296-5p detected by luciferase reporter assay. **C** Effect of silencing or overexpressing MEG8 on miR-296-5p levels. **D** Expression of miR-296-5p in the serum of DVT patients and healthy individuals. **E** LncRNA MEG8 levels in serum of DVT patients were negatively correlated with miR-296-5p. ***P < 0.001

To investigate the mechanism of MEG8 regulation of DVT, we searched the database for downstream target genes of MEG8. miR-296-5p has binding sites with MEG8 as illustrated in Fig. 4A. The interactions between MEG8 and miR-296-5p were verified by dual luciferase reporter genes, and the results suggested that co-transfection of MEG8-WT with miR-296-5p inhibitor elevated luciferase activity, and co-transfection of MEG8-WT with miR-296-5p mimic reduced it, whereas there was no significant difference in co-transfection of MEG8-MUT with them (Fig. 4B). Moreover, silencing MEG8 elevated the miR-296-5p expression, while overexpression of MEG8 decreased the level (Fig. 4C). We further examined showed that miR-296-5p levels were lower in DVT patients than in healthy individuals (Fig. 4D). The findings of Pearson correlation analysis revealed that there was a negative correlation between miR-296-5p and MEG8 in the serum of DVT patients (*r* = −0.618, *P* < 0.001, Fig. 4E).

### Effect of MEG8 and miR-296-5p acting together on migration, viability, apoptosis and inflammatory cytokines in HUVECs

**Fig. 5:**
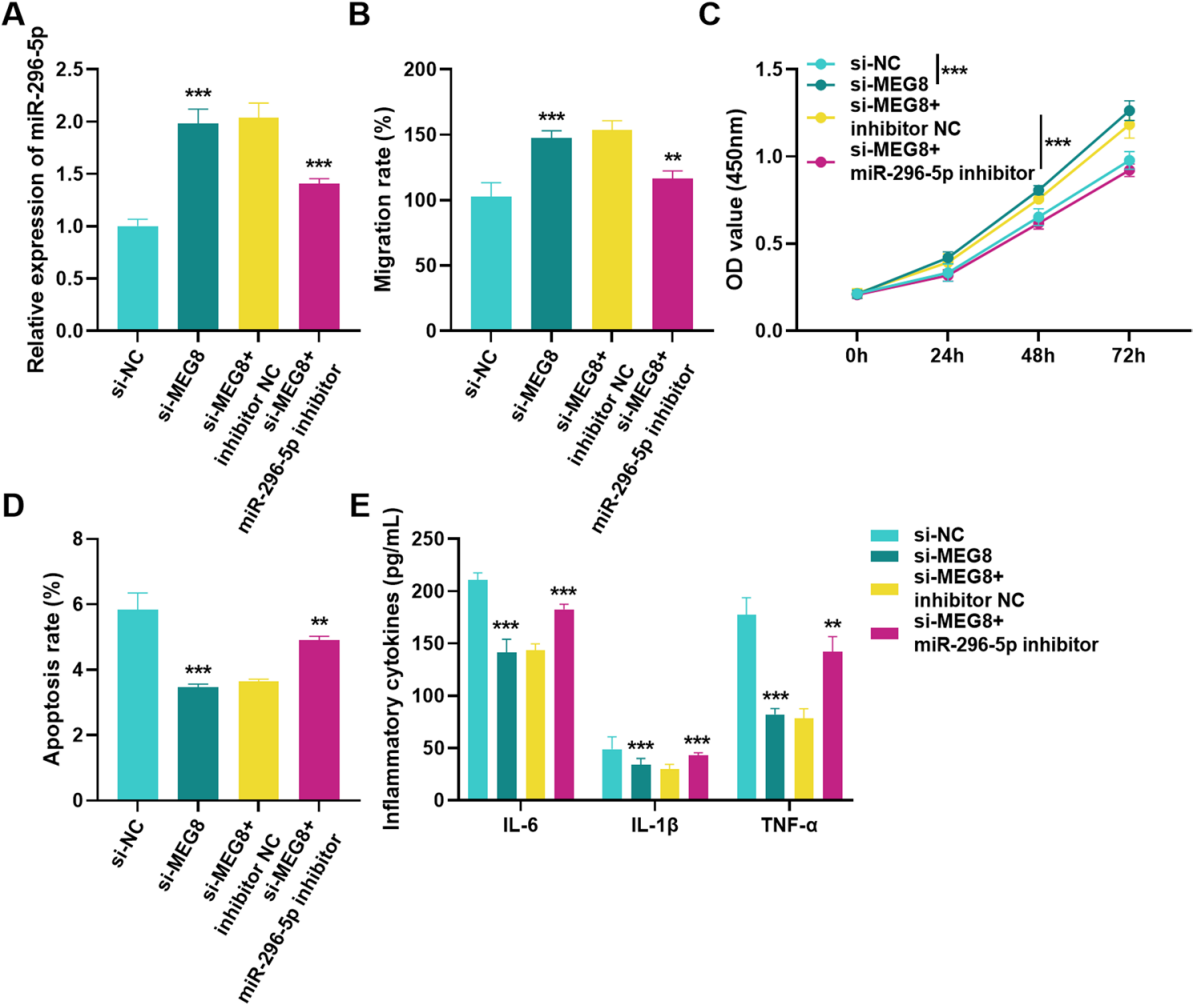
Effect of MEG8 and miR-296-5p acting together on HUVECs. Effect of co-transfection of si-MEG8 and miR-296-5p inhibitor on (**A**) miR-296-5p levels, (**B**) migration, (**C**) cell viability, (**D**) apoptosis, (**E**) IL-6, IL-1β and TNF-α in HUVECs. **P < 0.01, ***P < 0.001

To examine the regulatory function of MEG8 and miR-296-5p in DVT, we validated it by co-transfecting si-MEG8 and miR-296-5p inhibitor. RT-qPCR showed that co-transfection of si-MEG8 and miR-296-5p inhibitor decreased miR-296-5p levels compared to transfection of si-MEG8 (Fig. 5A). Cell migration and proliferation assays indicated that down-regulation of miR-296-5p reversed promotion of cell migration and proliferation by silencing MEG8 (Fig. 5B, C). Apoptosis experiments displayed that co-transfection of si-MEG8 and miR-296-5p inhibitor promoted apoptosis (Fig. 5D). Additionally, down-regulation of miR-296-5p reversed inhibition of pro-inflammatory factors by silencing MEG8 (Fig. 5E).

## Discussion

DVT is a common and tricky disease in vascular surgery, which may cause serious complications such as pulmonary embolism and post-thrombotic syndrome, endangering the life and health of patients [19, 20]. Therefore, the development of biologic therapies for DVT for early diagnosis and targeted intervention of DVT is essential. This study demonstrated that lncRNA MEG8 is remarkably overexpressed in DVT patients and exhibits superior clinical diagnostic ability. MEG8 overexpression inhibited the migration and viability of HUVECs and promoted apoptosis and inflammatory cytokines release. Furthermore, our study confirmed that MEG8 sponges miR-296-5p. Functional experiments suggested that miR-296-5p inhibitor reversed regulatory effects of MEG8 silencing on HUVECs. This finding enriched the knowledge of lncRNA regulatory network in thrombotic diseases.

HUVECs are involved in physiological and pathological processes such as angiogenesis, inflammation and oxidative stress [21, 22]. Furthermore, HUVECs exhibit significant stem cell characteristics, enabling them to accurately mimic the biological behavior of vascular endothelial cells in vivo, thereby providing a reliable model for investigating the molecular mechanisms associated with DVT [23, 24]. HUVECs have been selected in many DVT studies to examine the role of genes. For example, NEAT1/miR-218-5p/GAB2 axis and MALAT1/miR-383-5p/BCL2L11 axis may be involved in the formation of DVT by affecting the proliferation, migration and apoptosis of HUVECs [25, 26].

With continuous research, lncRNAs have shown an irreplaceable position for diagnosis and therapy of cardiovascular diseases. For instance, the lncRNA PSMB8-AS1 is markedly elevated in human atherosclerotic plaques and promotes vascular inflammation and atherosclerosis by regulating the NONO/PSMB9/ZEB1 axis, demonstrating that it is possible to develop lncRNA-based strategies to combat cardiovascular disease [27]. The lncRNA NUDT6 affects vascular disease progression by modulating the FGF2 and CSRP1 pathways, providing a novel RNA therapy target for vascular disease [28]. LncRNA CRNDE levels are elevated in DVT patients’ serum, and its combination with ultrasound shows high clinical value in the early diagnosis of women postpartum lower extremity DVT [29]. In addition, MEG8 showed a trend towards upregulation in ischemic stroke and affected disease progression by regulating vascular endothelial cell viability, migration and angiogenesis [16]. The migratory capacity of HUVECs is closely associated with the formation of DVT. In the initial stage of DVT, damage to venous endothelial cells is a core component, and cell migration is a key process for achieving repair following injury [30]. In DVT research, assessing the migration capacity of HUVECs and evaluating the dynamic changes in their reparative function may reflect the repair potential of cells. In our study, MEG8 levels were significantly elevated in DVT patients’ serum. In HUVECs, overexpression of MEG8 suppressed migration and viability, and facilitated apoptosis and inflammatory cytokines release, whereas silencing of MEG8 showed the opposite results. This is similar to the study published by Wang et al. on the role of lncRNA MALAT1 in DVT [26]. These results suggested that MEG8 may act in the pathogenesis of DVT by affecting cellular functions and inflammatory responses in HUVECs.

Several investigations have revealed that lncRNAs are involved in disease progression by sponging miRNAs and forming a lncRNA-miRNA regulatory network. For example, a review of the literature indicates that co-expression of lncRNAs with miRNAs regulates the biological functions of HUVECs, macrophages and cardiomyocytes [31]. In addition, lncRNA HOTAIR is upregulated in breast cancer tissues, and is a sponge for miR-129-5p as well as promotes breast cancer progression [32]. LncRNA XIST improves HMGB1 expression to exacerbate DVT through regulating ROS/NF-κB pathway through sponging of miR-103a-3p, which may be its potential therapeutic target [33]. Bioinformatics analysis revealed that MEG8 is involved in ovarian cancer progression by regulating miR-378d [34]. In our study, it was found that miR-296-5p has binding sites with MEG8 through target prediction. Previous studies have demonstrated that miR-296-5p is downregulated in thyroid cancer, atherosclerosis, and pulmonary hypertension and participates in the disease process [35–37]. In addition, miR-296-5p was identified in DVT studies as a possible diagnostic biomarker and therapeutic target [18]. Our study found that MEG8 is involved in the progression of DVT by sponging miR-296-5p. Down-regulation of miR-296-5p reversed effects of silencing MEG8 on cellular functions and inflammatory cytokines in HUVECs. These results indicated that MEG8 promoted DVT progression by sponging miR-296-5p to regulate HUVECs.

There are several limitations to this study. The downstream target genes of miR-296-5p have not been identified yet. In the future, transcriptome sequencing technology and bioinformatics will be used to screen the downstream targets in order to clarify the regulatory network of DVT. In addition, the validation of animal models is lacking, and subsequent experiments will construct the mouse model of MEG8 knockout for validation.

This study establishes the critical role of MEG8 in DVT development by sponging miR-296-5p, providing new perspectives for the molecular study of DVT. The research results create new avenues for early diagnosis and targeted treatment of DVT.

## Supplementary Information

Supplementary Material 1.

## Data Availability

The datasets used and/or analysed during the current study are available from the corresponding author on reasonable request.
